# Carotid artery aneurysm as the initial presentation of Behçet’s disease: A case report

**DOI:** 10.1016/j.jvscit.2025.102011

**Published:** 2025-10-15

**Authors:** Ahmed Bengrad, Abdennasser Laarouchi, Mohamed Ouhmich, Youssef Banana, Oussama Anane, Abdellah Rezziki, Adnane Benzirar, Omar El Mahi

**Affiliations:** Department of Vascular Surgery, Mohammed VI University Hospital of Oujda, Mohammed First University of Oujda, Oujda, Morocco

**Keywords:** Behçet’s disease, Carotid artery aneurysm, Case report

## Abstract

Behçet’s disease is a rare systemic vasculitis that may involve arteries and veins. Carotid artery aneurysms are extremely uncommon and can be the initial manifestation. We report a 48-year-old man presenting with a painless pulsatile cervical mass. Imaging revealed a right common carotid artery aneurysm, surgically resected with a reversed saphenous vein graft. Histopathology suggested vasculitis, and subsequent evaluation confirmed Behçet’s disease. This case highlights the importance of considering Behçet’s disease in isolated arterial aneurysms and the need for combined surgical and immunosuppressive therapy.

Behçet's disease is a chronic, relapsing, systemic vasculitis of unknown etiology, characterized by inflammatory involvement of the mucous membranes, skin, joints, eyes, and occasionally the central nervous system and vascular system. It is more commonly seen in young men from countries along the ancient Silk Road, extending from the Mediterranean to East Asia.^1^

Vascular involvement occurs in approximately 25% to 30% of cases and may affect both veins and arteries. However, arterial complications are less frequent, typically involving the aorta, pulmonary arteries, or femoral arteries. Arterial aneurysms represent a severe and potentially life-threatening manifestation of Behçet’s disease, due to the risk of rupture.^2^^,^^3^

Carotid artery aneurysms are exceptionally rare in Behçet’s disease. Diagnosis is often delayed due to the nonspecific nature of symptoms. These aneurysms may present as a pulsatile cervical mass, hoarseness, or may be discovered after ischemic or hemorrhagic complications. Management is based on vascular surgery combined with immunosuppressive therapy to control the underlying inflammation.^4^^,^^5^

We report a rare clinical case of a right common carotid artery aneurysm, revealed by a cervical mass in a previously healthy patient, ultimately diagnosed as Behçet’s disease. This case highlights the importance of considering systemic inflammatory conditions in the differential diagnosis of atypical arterial aneurysms in young or middle-aged patients.

## Case report

A 48-year-old man with no significant past medical history presented with a painless, pulsatile mass in the right cervical region, progressively enlarging over several weeks. He reported no associated symptoms such as pain, dysphagia, hoarseness, or neurological deficits. There was no history of recent trauma or infection.

On physical examination, a firm, pulsatile, nontender mass was palpable along the anterior border of the right sternocleidomastoid muscle. The overlying skin was normal, with no signs of inflammation, and no bruits were heard on auscultation.

**Fig 1:**
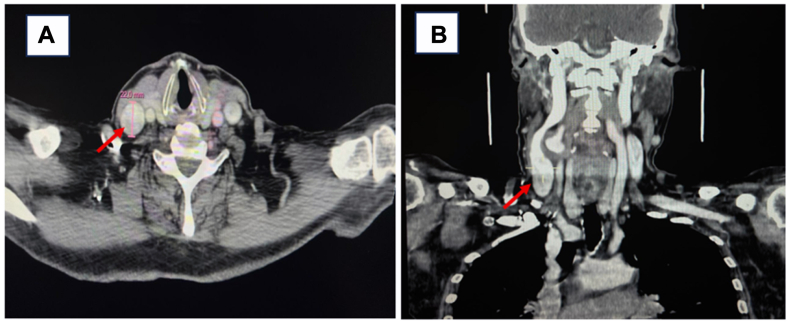
Cervical computed tomography angiography showing a saccular aneurysm of the right common carotid artery, measuring 22 mm, visible on axial **(A)** and coronal **(B)** views (*red arrows*).

An urgent contrast-enhanced computed tomography angiography of the neck revealed a saccular aneurysm of the right common carotid artery, measuring approximately 22 mm in diameter, with preserved distal flow and no evidence of dissection or thrombosis (Fig 1).

**Fig 2:**
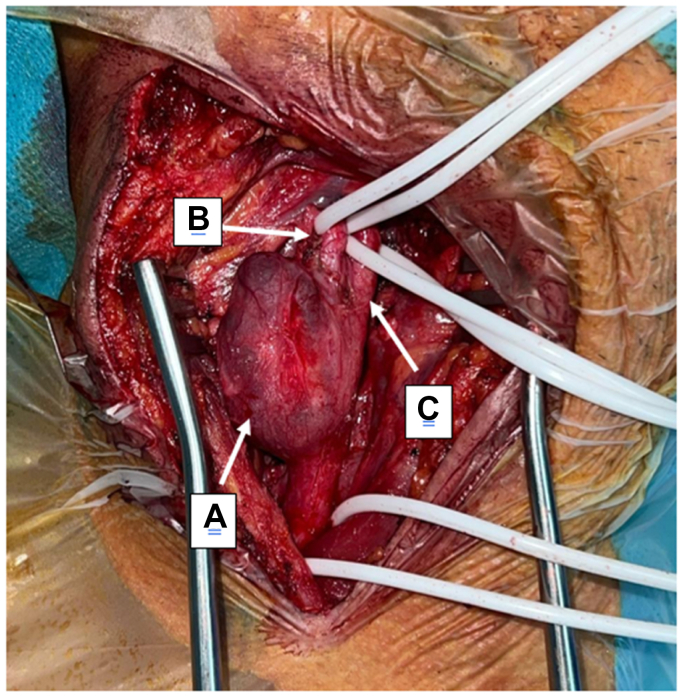
Intraoperative view of the carotid exposure showing a saccular aneurysm of the common carotid artery **(A)**, the internal carotid artery **(B)**, and the external carotid artery **(C)**.

**Fig 3:**
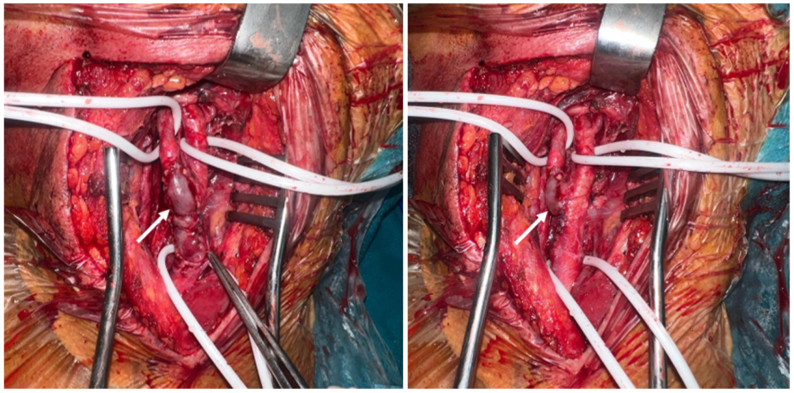
Intraoperative view showing aneurysm resection and restoration of arterial continuity with a reversed great saphenous vein graft (*white arrow*).

The patient underwent surgery under general anesthesia through a standard cervical approach (Fig 2). The aneurysm was carefully dissected and completely resected. Arterial continuity was restored with a reversed great saphenous vein graft harvested from the left lower limb (Fig 3). The procedure was uneventful.

The postoperative course was simple, with no neurological or vascular complications.

Histopathological examination of the aneurysmal wall revealed features of active vasculitis, including transmural inflammatory infiltrates and focal destruction of the elastic lamina.

Further clinical questioning revealed a history of recurrent oral aphthous ulcers and painful genital ulcers over the past year. In the absence of other underlying causes, Behçet’s disease was suspected according to the International Criteria for Behçet’s Disease. The patient was referred to the internal medicine department for specialized management and was started on immunosuppressive therapy, including corticosteroids and azathioprine, to control disease activity and prevent recurrence.

## Discussion

Arterial involvement in Behçet’s disease, although less frequent than venous manifestations, represents one of the most serious complications. It is associated with high morbidity and mortality, particularly in cases involving aneurysmal degeneration or major vessel rupture.^2^^,^^3^

Among the various arterial territories, the carotid arteries are rarely affected. Carotid aneurysms are exceptional in Behçet’s disease and may remain clinically silent or present as a painless pulsatile mass in the cervical region, as seen in our patient.^4^^,^^6^ In some cases, symptoms may arise from compression of adjacent structures, thromboembolism, or rupture, requiring urgent intervention.^7^

Histologically, these aneurysms result from transmural inflammation, leading to destruction of the media and elastic lamina, promoting vessel wall weakening and aneurysmal formation.^5^ This vasculitic process explains the high risk of recurrence or de novo aneurysm formation, especially in the absence of immunosuppressive therapy.

Open surgical repair is the preferred treatment in symptomatic or accessible arterial aneurysms. In Behçet’s disease, autologous grafts such as the reversed saphenous vein are generally favored due to their superior resistance to infection and thrombosis compared with prosthetic materials.^2^^,^^6^ In our case, surgery allowed for complete resection and histological confirmation of vasculitis, contributing to the etiological diagnosis.

Although endovascular techniques are increasingly used in vascular surgery, their application in patients with Behçet’s disease is controversial due to the risk of pseudoaneurysm formation at landing zones, stent migration, or endoleaks, particularly in an inflamed and fragile arterial wall.^8^

Beyond the surgical repair, medical management is essential. High-dose corticosteroids are the first-line treatment, often combined with immunosuppressive agents such as azathioprine, cyclophosphamide, or anti-tumor necrosis factor agents, especially in severe or recurrent vascular involvement.^1^^,^^9^ Early initiation of immunosuppression improves long-term vascular outcomes and reduces postoperative complications.^10^

In our case, the vascular event was the initial presentation of Behçet’s disease. Diagnosis was made retrospectively based on a targeted clinical history revealing recurrent oral and genital ulcers. This presentation is not uncommon; in some series, vascular lesions precede mucocutaneous signs in up to 10% to 15% of patients.^11^

This case highlights the importance of considering systemic inflammatory conditions such as Behçet’s disease in the differential diagnosis of isolated arterial aneurysms, particularly in young or middle-aged patients with no atherosclerotic risk factors. A multidisciplinary approach, including vascular surgery and internal medicine, is critical to ensure accurate diagnosis and appropriate long-term management.^12^

## Conclusions

Carotid artery aneurysms are rare vascular manifestations of Behçet’s disease and may represent its initial presentation. This case highlights the importance of considering systemic inflammatory disorders in young patients with atypical arterial aneurysms. Combined surgical and immunosuppressive treatment is essential to prevent recurrence and improve long-term outcomes.

## Funding

None.

## Disclosures

None.
